# Validation of markers for resistance to Pyrenophora teres f. teres loci on barley chromosomes 3H, 4H, and 6H in the polygenic inheritance of the trait

**DOI:** 10.18699/vjgb-25-133

**Published:** 2025-12

**Authors:** O.S. Afanasenko, N.V. Mironenko, N.M. Lashina, I.V. Rozanova, E.I. Kyrova, Yu.S. Nikolskaya, A.A. Zubkovich

**Affiliations:** All-Russian Research Institute of Plant Protection (VIZR), St. Petersburg, Russia; All-Russian Research Institute of Plant Protection (VIZR), St. Petersburg, Russia; All-Russian Research Institute of Plant Protection (VIZR), St. Petersburg, Russia; Sirius University of Science and Technology, Center of Genetics and Life Sciences, Sochi, Russia; All-Russian Research Institute of Plant Protection (VIZR), St. Petersburg, Russia; All-Russian Research Institute of Plant Protection (VIZR), St. Petersburg, Russia; Research and Practical Center of Agriculture of the National Academy of Sciences of Belarus, Zhodino, Belarus

**Keywords:** barley, net blotch, landraces, resistance, SNP markers, CAPS markers, KASP markers, образцы ячменя, сетчатая пятнистость, устойчивость, SNP-маркеры, CAPS-маркеры, KASP-маркеры, доноры устойчивости

## Abstract

The causal agent of net blotch Pyrenophora teres Drechs. f. teres (Ptt) is a dangerous pathogen of barley. The development of genetic protection against this disease is a necessary link in resource-saving and environmentally friendly barley cultivation technologies. Effective QTL markers controlling both qualitative and quantitative resistance are required for breeding for resistance to Ptt. As a result of GWAS, we identified barley accessions of different origins, the SNP haplotypes of which were associated with resistance loci simultaneously on different barley chromosomes (VIR catalogue numbers: k-5900, k-8829, k-8877, k-14936, k-30341 and k-18552). The aim of the study was to validate SNP markers (MM) of Ptt resistance loci on chromosomes 3H, 4H and 6H in F2 from crossing six resistant accessions with the susceptible variety Tatum. The observed segregation for resistance in all crossing combinations confirmed the presence of several genetic determinants of resistance in the studied accessions. To study the polymorphism of the parents from the crosses and the correspondence between the phenotypes to the presence/absence of the markers in the segregating populations, primers with a specific 3’-end, CAPS markers, and KASP markers were developed. A significant association (p < 0.05) between the presence of the CAPS marker JHI-Hv50k-2016-391380 HindIII on chromosome 6H and the phenotype of resistance to Ptt in F2 plants was revealed in crosses between the susceptible cultivar Tatum and accessions k-5900, k-8829, k-8877 and k-18552. On chromosome 4H, a significant association with the resistance phenotype in the F2 population from the cross with accession k-8877 was revealed for marker JHI-Hv50k-2016-237924, and in that from the cross with accession k-5900, for marker SCRI_RS_181886. The presence of QTL on chromosome 6H, which controls qualitative resistance in four barley accessions, masks the expression of other genes, which explains the discrepancy between the resistance phenotype and the presence of molecular markers in the segregating populations. Resistance donors and molecular markers with proven efficacy can be used in marker-assisted selection (MAS) to develop barley cultivars resistant to net blotch

## Abstract (ru)

Возбудитель сетчатой пятнистости листьев ячменя Pyrenophora teres Drechs. f. teres (Ptt) относится к эпифитотийно опасным патогенам ячменя. Разработка генетической защиты от этой болезни – важное звено ресурсосберегающих и экологически безопасных технологий возделывания ячменя. Для селекции на устойчивость к Ptt необходимы эффективные маркеры QTL, контролирующих как качественную, так и количественную устойчивость. При проведении полногеномного анализа (GWAS) нами выявлены образцы ячменя различного происхождения, SNP-гаплотипы которых ассоциировались с локусами устойчивости одновременно на разных хромосомах. Целью исследований была валидация SNP-маркеров локусов устойчивости к Ptt на хромосомах 3Н, 4Н и 6Н с использованием F2 популяции от скрещивания шести устойчивых образцов к-5900, к-8829, к-8877, к-14936, к-30341 и к-18552 с восприимчивым сортом Tatum. Расщепление по устойчивости во всех комбинациях скрещиваний подтвердило наличие нескольких генетических детерминант устойчивости у изучаемых образцов. Для изучения полиморфизма родительских компонентов скрещиваний и соответствия фенотипа наличию/отсутствию маркера в расщепляющихся популяциях были разработаны праймеры со специфичным 3’-концом, CAPS- и KASP-маркеры. Значимая связь (p < 0.05) наличия CAPS- маркера JHI- Hv50k-2016-391380 HindIII на хромосоме 6Н и фенотипа устойчивости к Ptt у растений F2 выявлена в комбинациях скрещивания восприимчивого сорта Tatum с образцами к-5900, к-8829, к-8877 и к-18552; на хромосоме 4Н при фрагментном анализе значимая связь с фенотипом устойчивости в популяции F2 с участием образца к-8877 выявлена для маркера JHI-Hv50k-2016-237924, образца к-5900 для маркера SCRI_RS_181886 и образца к-8829 для маркера JHI-Hv50k-2016-166356. Наличие QTL на хромосоме 6Н, контролирующего качественную устойчивость у четырех образцов ячменя, маскирует проявление других генов с меньшим фенотипическим проявлением, что и является причиной несоответствия фенотипа устойчивости и наличия молекулярного маркера в расщепляющихся популяциях. Доноры устойчивости и молекулярные маркеры с доказанной эффективностью могут быть использованы в MAS для создания устойчивых к возбудителю сетчатой пятнистости сортов ячменя.

## Introduction

The causal agent of net blotch, Pyrenophora teres Drechs. f. teres (anamorph: Drechslera teres Sacc. (Shoem.) = Helminthosporium teres), is a dangerous pathogen of barley. Yield losses from this pathogen on susceptible cultivars under favorable conditions can reach 40 %, with annual losses estimated at 12–17 %. According to our data, the majority of both spring and winter barley cultivars registered in the State Register of Breeding Achievements are susceptible to the net blotch. This is partly due to the difficulties of working with hemibiotrophic pathogens: the strong dependence of resistance expression on environmental factors, incomplete dominance of resistance and, consequently, difficulties in selection in segregating hybrid populations, complex inheritance of resistance traits determined by multiple QTLs, and epistatic interactions between resistance genes.

Genetically protected cultivars are an essential component of resource-saving and environmentally friendly agricultural crop cultivation technologies. The development of effective genetic protection is based on the availability of genetically diverse donors of qualitative and quantitative resistance genes and their rational use, taking into account the ranges of pathogen populations in different climatic regions. Timely rotation of genetically protected cultivars helps stabilize the population composition of plant pathogens and reduce the likelihood of epidemics.

Currently, using biparental mapping populations and genome-wide association study (GWAS) technology, genes and loci for quantitative resistance (QTL) to P. teres f. teres (Ptt) have been identified on all barley chromosomes (Steffenson et al., 1996; Richter et al., 1998; Friesen et al., 2006; Manninen et al., 2006; Yun et al., 2006; Grewal et al., 2008, 2012; Gupta et al., 2010; Cakir et al., 2011; Liu et al., 2011; König et al., 2013, 2014; O’Boyle et al., 2014; Afanasenko et al., 2015, 2022; Richards et al., 2017; Wonneberger et al., 2017; Amezrou et al., 2018; Martin et al., 2018; Dinglasan et al., 2019; Novakazi et al., 2019; Rozanova et al., 2019; Clare et al., 2021; Rehman et al., 2025). In our study, in a collection of 449 barley accessions, genotyped using the 50K Illumina SNP chip for 33,818 markers, 15 loci and 43 SNPs significantly associated with resistance to Ptt haplotypes were identified (Novakazi et al., 2019). As a result of this work, a group of resistant barley accessions was identified, the SNP haplotypes of which were associated with resistance loci simultaneously on different barley chromosomes, which apparently indicates the presence of several QTL and a possible additive effect. For example, in six resistant barley accessions included in this study, k-5900, k-8829, k-8877, k-14936, k-30341 (VIR catalogue numbers) and k-18552 (cultivar Zolo), SNP marker haplotypes in each accession were associated with 5–8 resistance loci on chromosomes 3H, 4H, 6H and 7H.

The molecular markers (MMs) of genes and QTLs for resistance to P. teres f. teres identified in these studies and in the studies of other authors, in most cases, have not been validated in other genetic environments for their effective use in barley breeding.

The aim of this study was to validate the SNP markers for Ptt resistance loci on chromosomes 3H, 4H, and 6H, known from the scientific literature, in F2 populations obtained from crossing six resistant accessions with the susceptible cultivar Tatum.

## Materials and methods

Barley genotypes. Six resistant barley accessions were selected for crossing and obtaining segregating F2 populations (VIR catalogue numbers): k-5900, k-8829, k-8877, k-14936, k-18552 (Zolo cultivar), and k-30341. Their SNP marker haplotypes were associated with resistance loci on different barley chromosomes, including chromosomes 3H, 4H, and 6H. The productive two-row barley cultivar Tatum from Germany was used as the susceptible parent. The characteristics of the barley accessions are presented in Table 1.

**Table 1.**
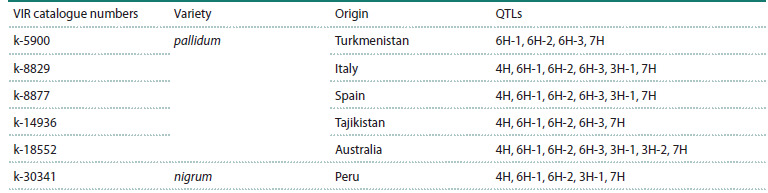
Origin of barley accessions and chromosomal location of QTLs associated with resistance (Novakazi et al., 2019) Note. Loci are within intervals determined using the Barleymap resource (https://barleymap.eead.csic.es/barleymap): 4Н – 58,942,545–67,692,302 bp and 448,603,913–449,611,912 bp, 6Н-1 – 64,219,990–67,138,358 bp, 6H-2 – 125,903,650–151,127,756 bp, 6Н-3 – 338,755,997–378,210,479 bp, 3H-1 – 119,627,830– 130,790,360 bp, 3H-2 – 490,244,247–491,381,651 bp, 7H – 5,165,127 bp. All barley samples had a row count of six.

P. teres f. teres isolates. Five Ptt isolates were used to assess resistance in a GWAS: No. 13 (Russia), Hoehenstedt (Germany), NFNB 50, NFNB 73, and NFNB 85 (Australia) (Novakazi et al., 2019). In this study, the resistance of these six accessions was assessed in addition to nine Ptt isolates of different origins (Table S1)1. For all isolates, the virulence formula was determined using a standard set of differentials (Afanasenko et al., 2009) (Table S2).

Supplementary Materials are available in the online version of the paper: https://vavilov.elpub.ru/jour/manager/files/Suppl_Afan_Engl_29_8.pdf


A study of barley resistance to P. teres f. teres. Methods for isolating the fungus into pure culture, storing it, grown on modified Chapek medium (KCL – 0.5 g, KH2PO4 – 0.5 g, MgSO4 – 0.5 g, urea – 1.2 g, lactose – 20 g, agar-agar – 20 g per 1 l of distilled water), and obtaining a Ptt conidial suspension for plant inoculation are described in detail in (Afanasenko et al., 2022; Lashina et al., 2023). The parent accessions and 65 seeds of each F1 hybrid population were sown in 1-liter containers with Terra Vita® potting soil. The plants were grown under controlled conditions in a VIZR climate room at 20–22 °C and a 16-hour photoperiod for 10–14 days. Barley plants were inoculated at the two- to three-leaf stage by spraying a suspension of single conidia isolates at a rate of 0.2 ml per plant. Conidia were counted with a hemocytometer, and the concentration was adjusted to 6,000 conidia/ml for inoculation. After inoculation, the plants were covered with plastic bags and left for 48 hours at 20–22 °C without light. After two days, the infected plants were transferred to light (TL-FITO VR LED lamps) with a 16-hour photoperiod and maintained at 60–70 % humidity.

Seedling response types were assessed on the second leaf 10–12 days after inoculation using a modified 10-point scale by A. Tekauz (1985), where values 1.0–4.9 indicated resistance; 5.0–5.9, an intermediate response; and 6.0–10, susceptibility.

Primer development. Three approaches were used to validate the identified SNP markers: a) Allele-Specific PCR (AS_PCR): development of primers with a 3′ end located at the position of the SNP of interest. Depending on the correspondence (complementarity) of the 3′ end SNP to the target DNA region, the presence or absence of a PCR amplification product is determined; b) Cleaved Amplified Polymorphic Sequences (CAPS): detection of SNPs using CAPS markers, the SNP of interest is located in the recognition site of a restriction endonuclease. As a result, the polymorphism of the restriction products determines the presence or absence of a restriction site in the amplicon – different genotypes will correspond to restriction fragments of different lengths in an agarose or polyacrylamide gel; c) Kompetitive allele-specific PCR (KASP): use of a PCR-based fluorescent genotyping system.

The candidate SNP position was confirmed using the Barleymap resource (https://barleymap.eead.csic.es/barleymap). The nucleotide sequences flanking the SNP (500 bp on each side) were exported to the Essembl Plants database (http:// plants.ensembl.org/index.html). Primer design was developed using the UGENE software package (v 49.1). For CAPS markers, the SnapGene Viewer software package (https://www. snapgene.com) was additionally used for sequence analysis and selection of a restriction endonuclease, differentiating genotypes based on the presence/absence of a restriction site at the SNP position.

To develop KASP markers, nucleotide sequences flanking the resistance-associated SNP (50 bp on each side) were exported from the Essembl Plants database (http://plants. ensembl.org/index.html). Based on these sequences, SNP allele-specific primer sequences were developed, using fluorescent tail sequences according to the protocol described by S. Jatayev et al. (2017).

DNA extraction and PCR conditions. DNA from frozen barley leaves was isolated using CTAB (cetyltrimethylammonium bromide). For this, the first leaf of each plant was ground in a mortar with liquid nitrogen supplemented with 2 % CTAB before inoculation with isolate F18. The homogenate was then lysed at 65 °C for two hours. DNA purification and extraction were performed according to the protocol (Murray, Thompson, 1980). The DNA precipitate was dissolved in deionized bidistilled sterile water to a concentration of 100–150 μg/μl. A C1000 thermal cycler (BIO-RAD) was used for amplification. The reaction was carried out in 25 μl: buffer (×10) – 2.5 μl, MgCl2 (50 mM) – 1.25 μl, dNTP (10 mM) – 0.5 μl, forward and reverse primers (10 pmol) – 0.25 μl each, Taq polymerase – 0.25 μl, water (bidistilled) – 19.0 μl, DNA (10–20 ng) – 1.0 μl. The optimal PCR conditions were selected for each primer. For most primers, the annealing temperature was 60 °C. Primers were purchased from Beagle (St. Petersburg). Restriction endonuclease digestion was performed according to the manufacturer’s protocols (SibEnzyme), and restriction products were visualized on a 2 % agarose gel (for HindIII, NruI, and RsaI).

Statistical data processing. Statistical analysis was performed using the χ2 test. Calculations were performed using STATISTICA 13.0 (Statsoft, www.statsoft.com) and the methodology described in N. Pandis (2016). For p < 0.05, Fisher’s exact test was additionally applied to the χ2 test

The diagnostic efficacy of the tested markers was determined as the ratio of the sum of true positive and true negative results to the total number of plants tested.

## Results

**Resistance of parental accessions**

The resistance of parental accessions to nine isolates of different origins, belonging to eight Ptt pathotypes, was studied (Table S2). All barley accessions exhibited race-specific resistance (Table 2). Of the nine isolates studied, one was virulent against k-8829, k-8877, k-14936, and k-18552, while four isolates were virulent against accession k-30341.

**Table 2.**
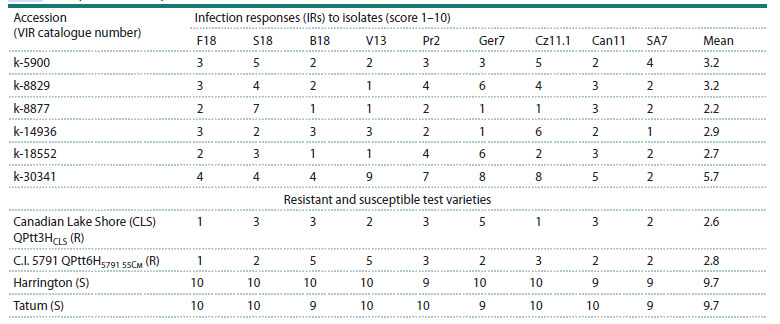
Response of barley accessions to inoculation with P. teres f. teres isolates

To analyze the segregation of resistance in F2 hybrid populations from the crossing of resistant barley accessions with the susceptible cultivar Tatum, the F18 isolate was used, since all the studied accessions were resistant to it, and the cv. Tatum demonstrated the maximum type of reaction – 10 (susceptibility) (see the Figure).

**Fig. 1.**
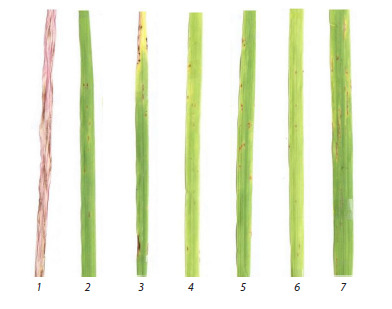
Types of reaction of parental accessions when infected with isolate F18, the damage score is indicated in brackets: 1 – Tatum (10), 2 – k-5900 (3), 3 – k-8829 (3.5), 4 – k-8877 (2), 5 – k-14936 (3), 6 – k-18552 (2), 7 – k-30341 (4).

**Segregation of resistance to Ptt in F2 populations from crosses of resistant barley accessions with the susceptible Tatum cultivar**

The results of segregation of resistance in F2 hybrid populations are presented in Table 3. The actual segregation in all cross combinations does not correspond to simple inheritance of the resistance, whether the class with intermediate reactions is combined with the class of resistant or susceptible plants, confirming the presence of multiple genetic determinants of resistance in the studied accessions (Table 3).

**Table 3.**
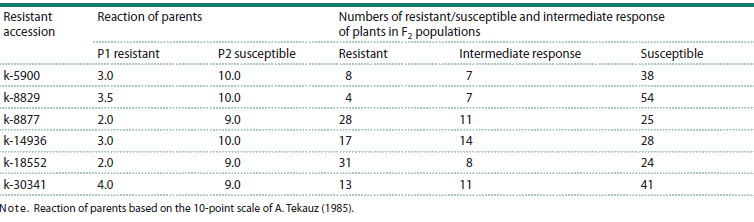
Segregation of resistance to isolate F18 in the F2 population from crossing resistant barley accessions with the susceptible cultivar Tatum Note. Reaction of parents based on the 10-point scale of A. Tekauz (1985

**Parental accession polymorphism for molecular markers on chromosome 4H**

To study the polymorphism of parental accessions, primers with a specific 3′ end (Table S3), CAPS markers, and competitive allele-specific PCR (KASP markers) were used. Ten markers on chromosome 4H were studied: five markers, identified from GWAS data, were associated with resistance to isolate No. 13 of P. teres f. teres in the position of 50.0–50.4 cM (Novakazi et al., 2019), and five markers were associated with Ptt resistance in the works of other researchers (Richards et al., 2017; Wonneberger et al., 2017; Amezrou et al., 2018). The positions of all 10 markers are listed in Table 4. CAPS markers were developed for two SNP markers on chromosome 4H.

**Table 4.**
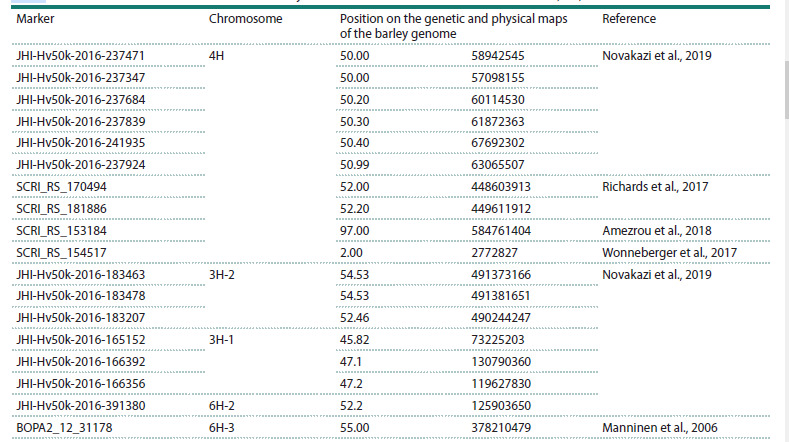
Positions of SNP markers associated with juvenile resistance to Ptt on chromosomes 4H, 3H, and 6H Note. The genetic position is determined from the current version of MorexV3 (Mascher et al., 2021).

Using the restriction endonuclease NruI for the JHIHv50k- 2016-237684 marker, two alleles are distinguished: in the presence of the T allele, which lacks a restriction site a 548 bp fragment is formed; in the presence of the C allele, which does have a restriction site, fragments of 197 bp and 351 bp are formed. Using the restriction endonuclease RsaI for the JHI-Hv50k-2016-237924 marker, two alleles can also be distinguished: the G allele is cut into fragments of 177, 105, 38, and 55 bp, and the C allele is cut into fragments of 177, 29, 76, 38, and 55 bp. Four SNP markers on chromosome 4H were converted to KASP marker format (Table S4).

**Fragment analysis**

The results of testing the developed primers on the parental accessions are presented in Table S5. The criterion for a promising marker was the presence of amplification products for resistant barley genotypes and the absence of them for susceptible ones, or vice versa. Polymorphism for the presence of amplification products in certain barley genotypes was detected for markers JHI-Hv50k-2016-237924 (4H-924), SCRI_RS_153184 (4H-184), and SCRI_RS_181886 (4H- 886). Figure S1 shows an example of polymorphism detection in the parental accessions using the SCRI_RS_181886 marker. The presence of the amplification product in both resistant and susceptible barley genotypes was detected using the primers of the remaining eight markers.

**Fragment length analysis of marker amplification products after restriction enzyme treatment**

Restriction analysis of the amplification products revealed polymorphism for markers JHI-Hv50k-2016-237684 and JHI-Hv50k-2016-237924 (Table 5): marker JHIHv50k- 2016-237684 (NruI restriction enzyme): two fragments (351 and 197 bp) were detected in four resistant accessions – k-8829, k-8877, k-14936, and k-30341. The amplification product of the susceptible Tatum cultivar and accessions k-5900 and k-18552 was 548 bp (Fig. S2); marker JHIHv50k- 2016-237924 (restriction enzyme RsaI): five fragments were detected in resistant accessions k-8829, k-8877, k-14936, and k-30341, while four fragments were detected in the susceptible cultivar Tatum and accessions k-5900 and k-18552 (Fig. S2). Thus, to study the co-segregation of MM and the resistance trait in segregating barley populations, markers JHI-Hv50k-2016-237684 and JHI-Hv50k-2016-237924 and the corresponding restriction enzymes NruI and RsaI were used to digest the amplification products of both markers.

**Table 5.**
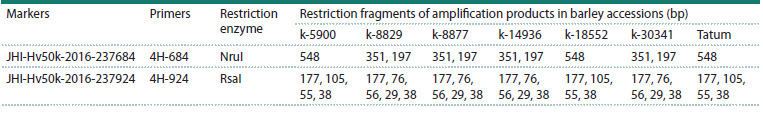
Results of detection of polymorphic restriction fragments of marker amplification products on chromosome 4H in parental accession

The results of the study of polymorphism for KASP markers on chromosome 4H of the parental accessions are presented in Table 6. Allelic polymorphism of resistant samples and the susceptible variety Tatum was detected for four markers: JHI-Hv50k-2016-237471 (4H-471), JHI-Hv50k-2016-237839 (4H-839), JHI-Hv50k-2016-241935 (4H-935), and JHIHv50k- 2016-237347 (4H-347), which were used to study the co-segregation of the resistance phenotype and the marker genotype

**Table 6.**
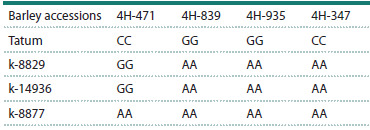
Polymorphism of KASP markers on chromosome 4H in parental accessions

**Molecular markers polymorphism on chromosome 3H in parental accessions used for crossing**

According to GWAS data, resistance in accessions k-8829, k-8877, k-18552, and k-30341 was also associated with the 3H-1 and 3H-2 loci (Tables 1 and 4). We previously validated KASP markers for these loci on chromosome 3H in segregating populations, which were highly effective (over 80 %) in the CLS, Morex, and Fox barley genotypes carrying the major resistance gene qPttCLS (Afanasenko et al., 2022). These KASP markers in intervals 45.82–47.2 and 52.46–54.53 cM were used to analyze segregating populations obtained from crossing the Tatum cultivar with Ptt-resistant accessions (Table S6).

For fragment analysis of marker amplification products on chromosome 3H, the primers proposed in the article by O. Afanasenko et al. (2022) were also used (Table S7). In fragment analysis, polymorphism for the presence of amplification products in three resistant accessions (k-8877, k-5900, and k-8829) and the susceptible Tatum cultivar was detected only for the JHI-Hv50k-2016-166356 marker, which was used to analyze the segregating populations. For the remaining six markers, no polymorphism was observed between the resistant accessions and the susceptible cultivar Tatum.

When using KASP markers, polymorphism for SNP haplotypes was detected only for one resistant accession, k-14936 (GG), and the susceptible cultivar Tatum (CC), and only for marker JHI-Hv50k 2016-165152. KASP markers JHI-Hv50k-2016-166392, JHI-Hv50k-2016-183463, and JHI-Hv50k-2016-183207 exhibited heterozygous SNP haplotypes, making them unsuitable for labeling accessions (Table S8).

**Parental accession polymorphism on chromosome 6H**

Resistance in the studied accessions was also associated with several loci on chromosome 6H (Tables 1 and 4). Previously, using double haploid mapping populations, the major RPt5 gene, determining high-quality resistance to Ptt, was identified on chromosome 6H in the position 52.00–55.03 cM in barley accessions CI9819, CI5791, and k-23874 (Manninen et al., 2006; Potokina et al., 2010; Koladia et al., 2017). The GWAS results (Novakazi et al., 2019) confirmed the presence of resistance loci in this interval, the markers of which were combined into four groups, depending on their location on the genetic and physical maps of barley (Tables 1 and 4). In previous studies, using barley accessions CI9819, CI5791 and k-23874 as tester genotypes, we demonstrated the effectiveness of two markers of resistance loci on chromosome 6H, which were used in this study (Table 4): JHI-Hv50k-2016-391380 (6H-380) at position 52.20 (6H-2) and BOPA2_12_31178 (6H-178) at position 55.03 cM (6H-3) (unpublished data). A CAPS marker was developed using the 6H-380 marker using the HindIII restriction enzyme. Allele A: restriction site → two fragments of 282 and 254 bp; allele G: undigested fragment of 536 bp

Primers for markers associated with resistance to P. teres f. teres on chromosome 6H are given in Table S9. Both markers showed polymorphism for the parental accessions. Marker 6H-380: HindIII restriction enzyme did not digest the amplification product of marker 6H-380 in all six resistant genotypes (one fragment), but digested it in the susceptible cultivar Tatum (two fragments). Marker 6H-178 revealed polymorphism between resistant barley accessions k-5900, k-8829 and the susceptible cv. Tatum (Fig. S3).

**Study of co-segregation of resistance to Ptt and molecular markers in segregating populations**

To study co-segregation for resistance to Ptt and identified polymorphic markers on chromosomes 4H, 3H, and 6H, 10 resistant and 10 susceptible lines were selected in each hybrid population. In some cross combinations, the analyzed sample of hybrid plants was expanded to 40 (20 resistant and 20 susceptible) to confirm segregation results

**Fragment analysis using polymorphic molecular markers**

The results of the correlation between the F2 plant resistance phenotype and the presence/absence of MM amplification products are presented in Table 7. A significant association between the marker and plant resistance using the χ2 criterion was found for marker 3H-56 in the Tatum × k-8829 combination, but Fisher’s exact test did not confirm the significance of the association (Table 7).

**Table 7.**
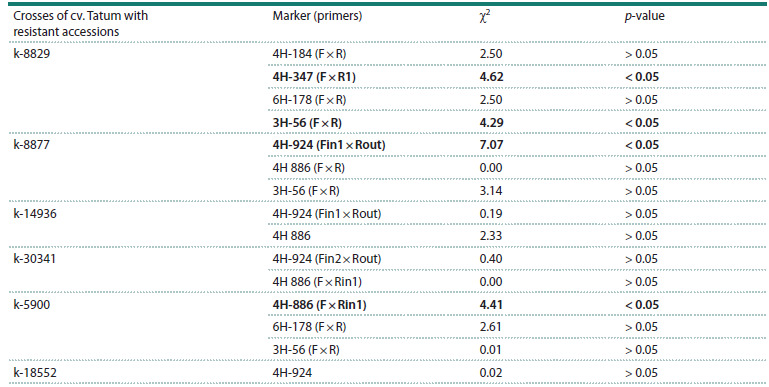
Reliability of the association between the Ptt resistance and molecular markers polymorphic on the parental accessions (fragment analysis) Note. The relationship between the resistance phenotype and the marker is significant at p < 0.05, highlighted in bold.

A significant association between the F2 plant resistance trait and marker 4H-924 was found in the Tatum × k-8877 cross, as well as with marker 4H-886 (F × Rin1) in the Tatum × k-5900. For the remaining MMs studied, despite polymorphism in the parental crosses, no significant association with resistance was found in the segregating populations. The obtained data indicate the presence of a QTL for Ptt resistance on chromosome 4H in accessions k-8829, k-8877, and k-5900.

**Analysis of the correlation between the resistance phenotype of F2 plants and the restriction products of CAPS markers**

Two markers on chromosome 4H were found to be polymorphic in the sizes of restriction products in the parental components of the crosses: 4H-684 NruI and 4H-924 RsaI. Figures S4 and S5 demonstrate the polymorphism of the restriction fragments of marker 4H-684 by endonuclease NruI in the progeny of the crosses Tatum × k-8829 and Tatum × k-8877. No statistically significant association was found between the genotype and phenotype of disease resistance (p > 0.05) (Table S10). The significant association of features identified for the 4H-924 marker in fragment analysis was absent when using the CAPS marker 4H-924 RsaI.

On chromosome 6H, polymorphism in the sizes of restriction products was detected for the JHI-Hv50k-2016-391380 HindIII (6H-380 HindIII) marker in the susceptible cv. Tatum and the resistant accessions k-18552, k-8877, k-14936, k-8829, k-5900, and k-30341. A significant correspondence (p < 0.05) between the genotype and phenotype of resistance to Ptt in F2 plants was found in the combinations Tatum × k-18552, Tatum × k-8877, Tatum × k-8829, and Tatum × k-5900 (Table 8, Figs. S6–S8). Thus, accessions k-18552, k-8877, k-8829, and k-5900 have a resistance QTL on chromosome 6H at position 52.2 cM.

**Table 8.**
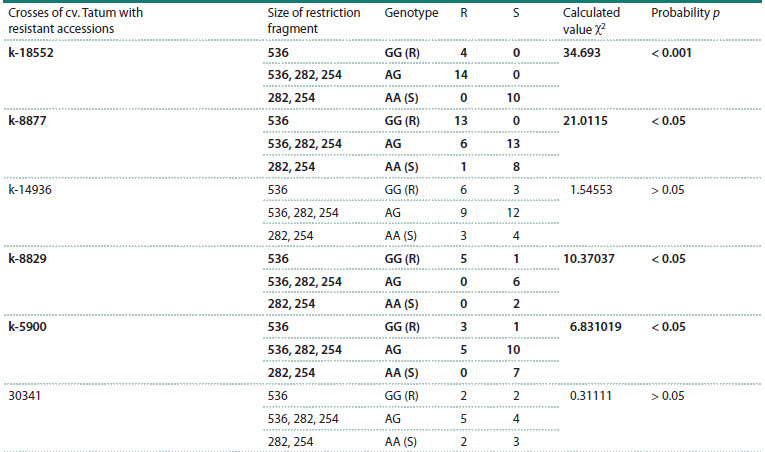
Correspondence between phenotypic resistance and restriction products of the CAPS marker JHI-Hv50k-2016-391380 (HindIII) on chromosome 6H in F2 from a cross between resistant accessions and the susceptible cv. Tatum Note. R – resistance, S – susceptibility. The association between the resistance phenotype and the marker is significant at p <0.05, highlighted in bold. The minimum table value of χ2 at significance level α of 0.05 was 5.991 for all barley samples.

KASP genotyping results

In three cross combinations, the resistant parents k-8829, k-14936, and k-8877 and the susceptible cv. Tatum were polymorphic for the SNP haplotypes of MM on chromosome 4H. In hybrid combinations involving the accession k-8829, the diagnostic efficiency was greater than 0.5 (0.6) for marker 4H-471 alone; for the remaining markers, this indicator was <q0.5. In the Tatum × k-8877 combination, the diagnostic efficiency of markers 4H-471, 4H-935, and 4H-347 was 0.71–0.73 (Table S11).

For the KASP marker JHI-Hv50k-2016-165152 on chromosome 3H, allelic polymorphism was detected in cv. Tatum (CC) and accession k-14936 (GG). In the segregating population from their cross, no correlation was found between plant resistance and haplotypes (Table S11).

A QTL on chromosome 6H, detected by the HindIII marker 6H380, determines high resistance to Ptt in four barley accessions and masks the presence of other QTLs (Tables S12–S14). Therefore, the resistance trait does not correlate with the other studied MMs. The absence/presence of MMs in susceptible plants of a particular hybrid combination is a different matter. For example, in the class of susceptible F2 plants in the Tatum × k-8877 combination, the homozygotes of the susceptible parent for the 4H-924 RsaI marker were 100 %, while for the 4H-684 NruI marker, six out of ten plants were homozygous and two heterozygous for susceptibility. Similar results were obtained for the KASP markers: for all four markers, six out of ten susceptible plants were homozygous for the susceptible parent’s allele, and three, heterozygous. In this cross, the four markers on chromosome 4H had a diagnostic efficiency of more than 0.7 (Table S12).

Susceptible plants predominated in the k-8829 × Tatum combination. The resistance phenotype split was 4 (R):7 (MR):54 (S), so only these four resistant plants and ten susceptible plants were included in the analysis. For the CAPS markers 6H380 HindIII and 4H-684NruI, as well as the KASP marker 4H-471, all heterozygous plants were associated with susceptibility, suggesting a recessive inheritance pattern. For the 4H-924RsaI marker, all susceptible plants had the genotype of the susceptible parent (Table S13).

In the k-5900 × Tatum combination, in addition to the proven significant correlation between the CAPS marker 6H380 HindIII and marker 4H-886 (F × Rin1), fragment analysis shows no obvious correspondence between the presence/ absence of markers 6H-178 and 3H-56 in the group of susceptible plants (Table S14).

## Discussion

Currently, 103 loci associated with juvenile and adult resistance to Ptt and a large number of MMs have been identified using GWAS technology and mapping in double haploid populations (Steffenson et al., 1996; Richter et al., 1998; Friesen et al., 2006; Manninen et al., 2006; Yun et al., 2006; Grewal et al., 2008, 2012; Cakir et al., 2011; Liu et al., 2011; Berger et al., 2013; König et al., 2013, 2014; O’Boyle et al., 2014; Afanasenko et al., 2015, 2022; Wang et al., 2015; Koladia et al., 2017; Richards et al., 2017; Wonneberger et al., 2017; Amezrou et al., 2018; Martin et al., 2018; Dinglasan et al., 2019; Novakazi et al., 2019; Rozanova et al., 2019; Rehman et al., 2025). However, there are only a few publications presenting the results of validation of Ptt resistance QTL markers identified in GWAS in a different genetic background (Grewal et al., 2010; Afanasenko et al., 2022).

Breeding barley for resistance to Ptt requires effective QTL markers controlling both qualitative and quantitative resistance. To validate the SNP markers of Ptt resistance loci on chromosomes 3H, 4H, and 6H identified in GWAS (Richards et al., 2017; Amezrou et al., 2018; Novakazi et al., 2019), barley accessions, the SNP haplotypes of which were associated with several Ptt resistance loci (Table 1), were selected. These accessions were resistant to a wide range of Ptt pathotypes in the juvenile phase (Table 2) and against a provocative background (late sowing) in the adult plant phase in the field (unpublished data).

Analysis of segregation for juvenile resistance in F2 from crosses of these accessions with the susceptible cultivar Tatum indicated complex inheritance of the trait, confirming the GWAS results. A distinctive feature of resistance assessment in segregating populations to Ptt, as well as to other hemibiotrophic pathogens, is the presence of a group of plants with intermediate reactions (scores 5.0–5.9). In the presence of several QTLs in the parental components of the cross, intermediate plant reactions are due to the presence of recombinants with different numbers of genetic determinants of resistance and different gene interactions.

Several Ptt resistance loci are known on chromosome 4H in the following intervals: 1.13 cM (Grewal et al., 2008); 3.31 cM (Afanasenko et al., 2015; Wonneberger et al., 2017); 47.27–52.69 cM (Richards et al., 2017; Novakazi et al., 2019); 64.3 cM (Steffenson et al., 1996); 77.0 cM (Martin et al., 2018); 97.66 cM (Amezrou et al., 2018); 113.1 cM (Martin et al., 2018); 121–123 cM (König et al., 2014); 150–175 cM (Friesen et al., 2006).

In this study, we examined markers of a locus located on chromosome 4H in the position of 50.0–50.4 cM, which we previously identified as a result of GWAS (Novakazi et al., 2019), as well as markers of loci identified by other researchers in the range of 52–52.2 cM (Richards et al., 2017), 97.0– 97.20 cM (Amezrou et al., 2018) and at the 2.0 cM position (Wonneberger et al., 2017). The choice of MM for studying co-segregation in segregating populations was based on the correlation of certain SNP haplotypes of markers identified in GWAS with the resistance phenotype. So, four “peak” markers associated with resistance to Ptt isolate No. 13 were identified on chromosome 4H in 98 barley accessions (average damage score of 3.54) (Table 9). The CCAT SNP haplotypes of these four markers were associated with resistance, while the GTGC SNP haplotypes of the same markers were associated with susceptibility (average damage score of 5.45) in 347 barley accessions (data kindly provided by F. Novakazi). However, among the 98 accessions, 16 with the CCAT haplotype were susceptible to the pathogen, and among the 347 accessions, 147 were resistant, although they had the GTGC haplotype. These data indicate that, despite the association of certain SNP marker haplotypes with resistance, random combinations of the same SNP haplotypes in susceptible accessions and vice versa are possible, which suggests the possibility of a false assumption about the presence of resistance-associated loci in certain accessions identified in GWAS.

**Table 9.**
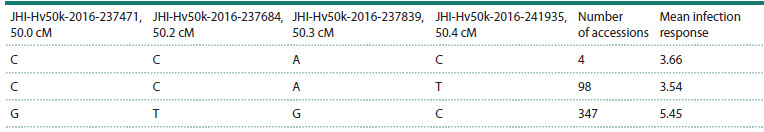
Mean infection responses of barley accessions with defined SNP haplotypes of four markers on chromosome 4H after inoculation with isolate P. teres f. teres No. 13

For most of the studied resistance loci markers on chromosomes 3H, 4H, and 6H, no polymorphism was detected in the MMs between the parental components of the cross (resistant accession × susceptible cultivar Tatum).

Of the 10 markers and 28 different primer combinations of these markers on chromosome 4H, only three were polymorphic in fragment PCR analysis, two were polymorphic when used as CAPS markers, and one KASP marker was polymorphic between the parental accessions in only one combination. A significant association between the F2 plant resistance trait and marker 4H-924 was found in the Tatum × k-8877 cross, as well as with marker 4H-886 (F × Rin1) in the Tatum × k-5900 cross. The obtained data indicate the possibility of using these markers in breeding if the k-8877 and k-5900 accessions are used as donors of resistance to Ptt.

In previous studies, determining the effectiveness of SNP markers of the resistance locus on chromosome 3H in the interval 46.29–54.3 cM using KASP genotyping revealed five markers that were 100–80 % effective in the double haploid population and in two segregating populations and were associated with resistance in the CLS, Morex, Fox cultivars, and accession k-21578 (Afanasenko et al., 2022). It was shown that the resistance locus on chromosome 3H contains at least two QTLs controlling resistance to Ptt in the inter- vals of 46.0–48.44 cM and 51.27–54.8 cM (Afanasenko et al., 2022). In this study, the same markers, located in the positions of 45.82–47.2 cM (3H-1) and 52.46–54.53 cM (3H-2), were used to examine segregating barley populations (Table 4). Of the seven primer–marker pairs studied on chromosome 3H, only one marker, JHI-Hv50k-2016-166356 (3H-56), detected polymorphism in five resistant accessions with the Tatum cultivar. However, only one cross, Tatum × k-8829, revealed a significant association between the marker and plant resistance. No correlation was found between these markers and the resistance phenotype using KASP geno- typing.

Resistance in the studied accessions was also associated with several loci on chromosome 6H. Previously, using double haploid mapping populations on chromosome 6H in the interval of 52.00–55.03 cM in barley accessions CI9819, CI5791, and k-23874, a large RPt5 gene was identified that determines high-quality resistance to Ptt (Manninen et al., 2006; Potokina et al., 2010; Koladia et al., 2017). As a result of GWAS (Novakazi et al., 2019), resistance loci were also identified in this position, the markers of which were combined into four groups, depending on their location on the genetic and physical maps of barley (Table 4). Previously, using barley accessions CI9819, CI5791 and k-23874 as test genotypes, we demonstrated the effectiveness of two markers of resistance loci on chromosome 6H, which were used in this study: JHI-Hv50k-2016-391380 HindIII (6H-380) at position 52.20 (6H-2) and BOPA2_12_31178 (6H-178) at position 55.03 cM (6H-3) (unpublished data).

A significant association (p < 0.05) between the JHIHv50k- 2016-391380 HindIII (6H-380) marker and the Ptt resistance phenotype in F2 plants was found in combinations from crossing the susceptible cv. Tatum with the k-5900 (Turkmenistan), k-8829 (Italy), k-8877 (Spain), and k-18552 (Australia) accessions. These data indicate the possibility of using these accessions as resistance donors and the CAPS marker JHI-Hv50k-2016-391380 HindIII in marker-assisted selection (MAS).

It is known that QTLs on chromosome 6H at the studied locus control high resistance in barley genotypes (Afanasenko et al., 1998; Manninen et al., 2006; Koladia et al., 2017). The presence of a highly significant association of the resistance phenotype of F2 plants in four cross combinations involving the k-5900, k-8829, k-8877, and k-18552 accessions of the SNP haplotype of the 6H-380 HindIII marker masks the presence of other QTLs. However, in the class of susceptible plants in a given cross combination, the markers must correspond to the genotype of the susceptible parent. For example, the KASP markers 4H-471, 4H-347, and 4H-935 and the CAPS marker 4H-924 RsaI, in combination with k-8877, can be effective for culling susceptible plants. However, when using the entire plant accession, no significant correlation was found between the resistance phenotypes and the genotypes of these markers

## Conclusion

Therefore, the absence or presence of amplification products of a polymorphic marker on the parental components of a cross in resistant F2 plants with polygenic inheritance does not prove that there is no correlation between the marker and the resistance trait, as the presence of a major resistance gene masks the expression of other QTLs

New donors of resistance to Ptt were identified: accessions k-5900 (Turkmenistan), k-8829 (Italy), k-8877 (Spain) and k-18552 (Australia), in which the QTL on chromosome 6H is located at position 52.2 cM, 125,903,650 bp. Accessions k-8877 and k-5900 also have a QTL on chromosome 4H in the position of 50.00–50.99 cM, 57,098,155–63,065,507 bp, and accession k-8829 has a QTL on chromosome 3H at position 47.2 cM, 1,196,27,830 bp. Resistance donors and validated MMs with proven efficacy can be used in MAS to develop barley cultivars resistant to net blotch.

## Conflict of interest

The authors declare no conflict of interest.
